# Evaluation of ^99m^Tc-Z_IGF1R:4551_-GGGC affibody molecule, a new probe for imaging of insulin-like growth factor type 1 receptor expression

**DOI:** 10.1007/s00726-014-1859-z

**Published:** 2014-11-27

**Authors:** Bogdan Mitran, Mohamed Altai, Camilla Hofström, Hadis Honarvar, Mattias Sandström, Anna Orlova, Vladimir Tolmachev, Torbjörn Gräslund

**Affiliations:** 1 Preclinical PET Platform, Department of Medicinal Chemistry, Uppsala University, Uppsala, Sweden; 2 Unit of Biomedical Radiation Sciences, Department of Radiology, Oncology, and Radiation Sciences, Rudbeck Laboratory, Uppsala University, 751 85 Uppsala, Sweden; 3 Division of Protein Technology, School of Biotechnology, KTH Royal Institute of Technology, Stockholm, Sweden; 4 Nuclear Medicine and PET, Department of Radiology, Oncology, and Radiation Sciences, Uppsala University, Uppsala, Sweden

**Keywords:** Affibody molecule, IGF-1R, Peptide-based chelator, ^99m^Tc, Biodistribution

## Abstract

Overexpression of insulin-like growth factor-1 receptor (IGF-1R) in several cancers is associated with resistance to therapy. Radionuclide molecular imaging of IGF-1R expression in tumors may help in selecting the patients that will potentially respond to IGF-1R-targeted therapy. Affibody molecules are small (7 kDa) non-immunoglobulin-based scaffold proteins that are well-suited probes for radionuclide imaging. The aim of this study was the evaluation of an anti-IGF-1R affibody molecule labeled with technetium-99m using cysteine-containing peptide-based chelator GGGC at C-terminus. Z_IGF1R:4551_-GGGC was efficiently and stably labeled with technetium-99m (radiochemical yield 97 ± 3 %). ^99m^Tc-Z_IGF1R:4551_-GGGC demonstrated specific binding to IGF-1R-expressing DU-145 (prostate cancer) and MCF-7 (breast cancer) cell lines and slow internalization in vitro. The tumor-targeting properties were studied in BALB/c nu/nu mice bearing DU-145 and MCF-7 xenografts. [^99m^Tc(CO)_3_]^+^-(HE)_3_-Z_IGF1R:4551_ was used for comparison. The biodistribution study demonstrated high tumor-to-blood ratios (6.2 ± 0.9 and 6.9 ± 1.0, for DU-145 and MCF-7, respectively, at 4 h after injection). Renal radioactivity concentration was 16-fold lower for ^99m^Tc-Z_IGF1R:4551_-GGGC than for [^99m^Tc(CO)_3_]^+^-(HE)_3_-Z_IGF1R:4551_ at 4 h after injection. However, the liver uptake of ^99m^Tc-Z_IGF1R:4551_-GGGC was 1.2- to 2-fold higher in comparison with [^99m^Tc(CO)_3_]^+^-(HE)_3_-Z_IGF1R:4551_. A possible reason for the elevated hepatic uptake of ^99m^Tc-Z_IGF1R:4551_-GGGC is a high lipophilicity of amino acids in the binding site of Z_IGF1R:4551_, which is not compensated in ^99m^Tc-Z_IGF1R:4551_-GGGC. In conclusion, ^99m^Tc-Z_IGF1R:4551_-GGGC can visualize the IGF-1R expression in human tumor xenografts and provides low retention of radioactivity in kidneys. Further development of this imaging agent should include molecular design aimed at reducing the hepatic uptake.

## Introduction

The insulin-like growth factor-1 receptor (IGF-1R) is a dimeric transmembrane tyrosine kinase receptor, which is activated by two ligands, insulin-like growth factor-1 (IGF-1) or insulin-like growth factor-2 (IGF-2). Binding of IGF-1 and IGF-2 is followed by signaling through several different intracellular pathways, leading to an increased rate of cellular proliferation and unresponsiveness to apoptotic signals (Dearth et al. 2007; Chitnis et al. 2008; Baserga 2009).

For several types of cancer, an abnormally high expression level of IGF-1R is associated with metastasis and resistance to therapy (Pollak et al. 2004; Werner and Bruchim 2009; LeRoith and Roberts 2003). Therefore, IGF-1R is considered as a therapeutic target in the treatment of breast (Guvakova and Surmacz 1997; Turner et al. 1997), prostate (Hellawell et al. 2002), lung (Ouban et al. 2003), colorectal (Hakam et al. 1999), endometrial and bladder carcinomas, melanomas, and sarcomas (Khandwala et al. 2000). The main therapeutic approaches directed against IGF-1R signaling include the use of anti-receptor monoclonal antibodies, ligand-neutralizing monoclonal antibodies, and tyrosine kinase inhibitors (Pollak 2012; Haisa 2013; Karamouzis and Papavassiliou 2012; Sachdev and Yee 2007). All these approaches are highly specific, and the success depends on pre-selecting the patients that have IGF-1R-expressing tumors and would therefore most likely respond to IGF-1R-therapies (Carden et al. 2009). Of equal importance is to exclude the potential non-responders in order to avoid overtreatment. Therefore, robust methods to determine IGF-1R expression status, both initially and as a consequence of treatment are necessary.

Currently, the most common and established method for detection of molecular targets in tumors is biopsy sampling followed by immunohistochemistry, enzyme-linked immunosorbent assay (ELISA), fluorescent in situ hybridization (FISH), or other assays. A drawback with these methods is that expression heterogeneity in the tumor might lead to false-negative findings. Moreover, the IGF-1R expression might change during the course of disease, which might necessitate multiple biopsies.

Radionuclide molecular imaging of molecular target expression allows repeated non-invasive investigation of receptor status at different time points and reduces possibility of errors caused by target expression heterogeneity (Tolmachev et al. 2010a). However, radionuclide molecular imaging of IGF-1R is challenging because of its expression in normal tissues and the relatively low expression level usually found in tumors (10,000–30,000 receptors per cell). The use of radiolabeled therapeutic anti-IGF-1R monoclonal antibodies is a straightforward approach for the development of agents for the visualization of IGF-1R expression in tumors. A monoclonal antibody R1507, labeled with ^111^In or ^89^Zr, was found to allow imaging of IGF-1R-expressing SUM149 breast cancer xenografts in mice (Heskamp et al. 2010). The probe showed specific uptake in tumors, but the long blood residence time resulted in high background (tumor-to-blood ratio of 3.8 ± 0.3) and delayed imaging time (3–7 days p.i.) (Heskamp et al. 2010). Despite these limitations, it was found that ^111^In-labeled R1507 could predict the response to therapy in a bone sarcoma model (Fleuren et al. 2011). The use of F(ab′)_2_ and Fab fragments of R1507 demonstrated that a higher imaging contrast can be achieved at earlier time points (Heskamp et al. 2012; Fleuren et al. 2013). These studies suggested that a reduction of the size of the probe is a promising approach for improving contrast (and therefore, sensitivity) of IGF-1R imaging. An important precondition for successful imaging was retained high affinity of the targeting agent.

Affibody molecules are a class of non-immunoglobulin-based imaging agents that are characterized by small size, high affinity, and specificity for their intended target. They are engineered scaffold proteins derived from one of the independently folding domains of staphylococcal protein A (Nygren 2008; Löfblom et al. 2010). The affibody molecules are only 58 amino acids long (*M*_w_ 6–7 kDa), they are robust and devoid of cysteine residues in the framework. They typically fold into anti-parallel three-helix bundles. The affibody molecules are characterized by an efficient extravasation and rapid biodistribution, and their small size allows for a rapid clearance of unbound probe from blood and healthy tissues. For many targets, affibody molecules with high affinity (low nanomolar or subnanomolar) have been generated, which prevents rapid washout from the tumor (Ahlgren and Tolmachev 2010). Achieving high concentrations of tracer in the tumor and low concentrations in other tissues results in high imaging contrast, which is crucial for imaging sensitivity. An additional advantage of the rapid in vivo kinetics of affibody molecules is that short-lived radionuclides can be used and imaging can be performed shortly after injection (Ahlgren and Tolmachev 2010).

The feasibility of radionuclide molecular imaging of IGF-1R-expressing xenografted tumors was demonstrated earlier, using the ^111^In-H_6_-DOTA-Z_IGF1R:4551_ affibody molecule (Tolmachev et al. 2012a). The probe was found to crossreact with murine IGF-1R, which makes mice an adequate model reflecting all interactions of the imaging agent. Tumor visualization was possible shortly after injection (4–8 h). However, the study has shown a high uptake in liver and spleen. The aforementioned feasibility study was performed using a construct containing an N-terminal hexahistidine tag. Experience using HER2-targeting affibody molecules indicated the presence of hexahistidine tag at N-terminus to be associated with elevated hepatic uptake (Ahlgren et al. 2008, 2009), while the use of a glutamate-containing tag with the amino acid sequence HEHEHE permitted an appreciable reduction of radioactivity uptake in the liver (Tolmachev et al. 2010b; Hofström et al. 2011). We have shown that a high hydrophilicity and negative charge of the HEHEHE-tag play a major role in balancing a positive charge and elevated lipophilicity of [^99m^Tc(CO)_3_]^+^ and in reduction of hepatic uptake of [^99m^Tc(CO)_3_]^+^-labeled anti-HER2 affibody molecules (Hofström et al. 2013). Consequently, the introduction of a HEHEHE-tag at the N-terminus of the anti-IGF-1R affibody molecule resulted in a significantly lower liver uptake for [^99m^Tc(CO)_3_]^+^-(HE)_3_-Z_IGF1R:4551_ compared to ^111^In-H_6_-DOTA-Z_IGF1R:4551_ (Orlova et al. 2013). However, the radioactivity uptake in the liver remained higher than the uptake in tumor. Furthermore, renal retention of residualizing ^111^In-DOTA and [^99m^Tc(CO)_3_]^+^-HEHEHE labels was also high. Even though renal metastases are uncommon, high renal uptake might complicate the detection of metastases in the lumbar region.

Multiple studies have previously demonstrated that internalization of affibody molecules by cancer cells after binding to an overexpressed receptor is rather slow, 20–30 % of cell-bound tracer per day (Ahlgren et al. 2008, 2009; Wållberg et al. 2011; Tolmachev et al. 2012a; Orlova et al. 2013). The same pattern was also observed for IGF-1R-binding affibody molecules in previous studies (Tolmachev et al. 2012a; Orlova et al. 2013). For this reason, residualizing properties of labels are not critical for tumor uptake shortly after injection. On the opposite, interaction of affibody molecules with scavenger receptors in excretory organs (kidneys and liver) leads to rapid internalization, transfer into lysosomal compartment and degradation. Radiocatabolites of non-residualizing labels “leak” rapidly from hepatocytes and proximal tubuli cells, while residualizing labels got trapped intracellularly. As a result, retention of residualizing labels is much higher than retention of non-residualizing ones in liver and, especially, in kidneys (Hofström et al. 2011; Tolmachev et al. 2009). Thus, the use of non-residualizing labels may, in principle, reduce uptake of affibody-delivered radioactivity in excretory organs without compromising uptake in tumors.

Wållberg et al. (2011) demonstrated previously that the use of a GGGC sequence at the C-terminus of an anti-HER2 affibody molecule as a peptide-based chelator for ^99m^Tc provided an efficient labeling. The label was stable in murine plasma in vitro. High in vivo stability of ^99m^Tc-GGGC-complex was also confirmed by experiments with anti-HER2 affibody molecule (Wållberg et al. 2011), which demonstrated low uptake of radioactivity in stomach and salivary gland. In the case of complex instability, relased ^99m^Tc-pertechnetate accumulates in these organs (Zuckier et al. 2004). Importantly, it was found that ^99m^Tc-GGGC-complex is a non-residualizing label, providing a high uptake in the tumor but low uptake in liver and kidneys. However, due to the limited experience using affibody molecules, it was unknown if the findings for HER2-targeting affibody molecules can be directly translated to affibody molecules for imaging of other targets.

The goal of the current study was to evaluate if labeling of the anti-IGF-1R affibody molecule Z_IGF1R:4551_, containing the peptide-based chelator GGGC at the C-terminus, with ^99m^Tc would provide a reduction of radioactive uptake in the excretory organs, and thereby improve imaging contrast. The best previous variant, [^99m^Tc(CO)_3_]^+^-(HE)_3_-Z_IGF1R:4551_, was used for head-to-head comparison.

## Materials and methods

### Materials

^99m^Tc was obtained as pertechnetate (^99m^TcO_4_^−^) from an Ultra-Technekow™ DTE Technetium Generator (Covidien), using 0.9 % sterile sodium chloride (Covidien) as generator eluent. Buffers, including 0.05 M phosphate buffered saline pH 7.4, and PBS containing 2 % BSA, were produced in house. *α*-d-gluconic acid sodium salt and ethylenediaminetetraacetic acid (EDTA) were purchased from Sigma-Aldrich, tin(II) chloride dihydrate (SnCl_2_ × 2H_2_O) was obtained from Fluka Chemika. Human recombinant IGF-1R was purchased from BioVision Inc. ^125^I was purchased from PerkinElmer (Waltham, MA, USA). Organic solvents were purchased from Merck (Darmstadt, Germany). Chloramine-T (CAT) and sodium metabisulfite were from Sigma Chemical Company (St. Louis, MO). The measuring of the radioactivity was performed using an automated gamma spectrometer equipped with a 3-in. NaI(Tl) detector (1480 WIZARD; PerkinElmer). Yield and radiochemical purity of the labeled affibody constructs were analyzed using instant thin-layer chromatography (ITLC) strips (150-771 DARK GREEN, Tec-Control Chromatography strips from Biodex Medical Systems). NuPAGE 10 % Bis–Tris gels (Life Technologies) were used for analysis of conjugates and for in vitro stability studies. The distribution of radioactivity along ITLC strips and SDS-PAGE gels was measured on a Cyclone™ Storage Phosphor System (PerkinElmer). The binding affinity tests were performed using a LigandTracer Yellow instrument provided by Ridgeview Instruments AB. An unpaired *t* test was used to determine significant differences (*p* < 0.05) in all experiments.

### Protein production

The gene encoding Z_IGF1R:4551_ (Tolmachev et al. 2012a) was PCR amplified and inserted into the plasmid pET21a(+) (Novagen) using the restriction enzymes *Nde*I and *Hind*III. The oligonucleotides used gave an expression cassette with the amino acid sequence M-Z_IGF1R:4551_-GGGC under control of the T7-promoter. The protein was expressed in *Escherichia coli* strain BL21(DE3)(Novagen) at 37 °C after induction by Isopropyl *β*-d-1-thiogalactopyranoside to a final concentration of 1 mM at OD_600_ = 0.8. Cells were grown in a 500-mL shake flask culture with Tryptic soy broth (Merck) supplemented with 5 g/L yeast extract (Merck) as medium. Production was performed for 3 h, after which the cells were harvested by centrifugation and frozen. On the following day, the cell pellet was thawed and resuspended in 30 mL deionized water after which the cells were broken by sonication. Cell debris was pelleted by centrifugation and the cleared lysate was recovered and heat treated at 70 °C for 10 min after which the formed precipitate was removed by centrifugation. The lysate was subsequently filtered through a 0.45-μm filter; potentially formed disulphide bridges were reduced followed by separation by reversed-phase HPLC (RP-HPLC) as described (Orlova et al. 2013).

The purity of Z_IGF1R:4551_-GGGC was determined by RP-HPLC as previously described (Tolmachev et al. 2010b). Z_IGF1R:4551_-GGGC was analyzed by electrospray ionization mass spectrometry (ESI MS) to confirm its authenticity as previously described (Tolmachev et al. 2010b).

### Labeling and stability test

Site-specific radiolabeling with technetium-99 m was performed using lyophilized kits. Each kit contained 5 mg of sodium *a*-d-glucoheptonate dihydrate, 100 μg of edetate disodium (Na_2_EDTA), and 75 μg of tin(II)chloride dihydrate (SnCl_2_ × 2H_2_O) as has been optimized earlier for anti-HER2 affibody molecules by Ahlgren et al. (2010). The content of the freeze-dried kit was dissolved in 100 μL degassed PBS and added to the vial containing 100 μg Z_IGF1R:4551_-GGGC. After adding 100 μL ^99m^TcO_4_^−^, the reaction vial was filled with argon gas to protect the mixture from oxidation, then thoroughly vortexed and incubated for 2 h (95 °C). After cooling down for 15 min, the labeling yield was determined by ITLC. PBS was used as mobile phase for analysis of the labeling efficiency. In this system, the proteins and colloids stay on application point, while unbound radioactivity moves with the front. Purification was performed by size-exclusion chromatography using disposable NAP-5 columns (GE Healthcare), which were pre-equilibrated and eluted with PBS containing 2 % BSA. The stability in serum was analyzed by mixing murine serum (200 μL) with 10 μL freshly labeled ^99m^Tc-Z_IGF1R:4551_-GGGC. The samples were incubated for 1 h at 37 °C. As control, 10 μL freshly labeled ^99m^Tc-Z_IGF1R:4551_-GGGC was mixed with 200 μL PBS containing 2 % BSA and incubated for 1 h at r.t. All samples were analyzed by both ITLC and sodium dodecyl sulfate polyacrylamide gel electrophoresis (SDS-PAGE) using MES–SDS buffer at 210 V constant. A sample of ^99m^Tc-pertechnetate was used as reference standard for low molecular weight compounds.

For radioiodination of the natural ligand, 20 µg of IGF-1 (1 mg/mL in PBS) was mixed with 20 MBq of ^125^I, and the mixture additionally buffered with 40 µL PBS. The electrophilic iodination was initiated by adding 10 µg chloramine-T (1 mg/mL in PBS). After 1 min, the reaction was quenched by adding 20 µg sodium metabisulfite (1 mg/mL in PBS), and ^125^I-IGF-1 was purified by size-exclusion chromatography using disposable NAP-5 column.

### Cell culture

Human prostate cancer (DU-145) and breast cancer (MCF-7) cell lines (ATCC, purchased via LGC Promochem) were used for binding specificity and cellular processing studies. The IGF1R expression in these cell lines was documented earlier (Li et al. 2013; Turney et al. 2012). Roswell Park Memorial Institute medium (RPMI) was used to culture all cell lines. The cells were counted using an electronic cell counter (Beckman Coulter).

### In vitro characterization

The in vitro binding specificity tests were designed to determine if the binding of ^99m^Tc-Z_IGF1R:4551_-GGGC to IGF-1R-expressing cells (MCF-7 and DU-145) was saturable and therefore receptor mediated. A cross blocking of Z_IGF1R:4551_-GGGC and IGF-1 was evaluated. In the first set of experiments, saturation of ^125^I-IGF-1 binding was tested. For each cell line, a group of nine Petri dishes was used. Each Petri dish contained a cell monolayer of ca. 7 × 10^5^ cells/dish. Cells in three of the six Petri dishes were pre-saturated with 500 μL blocking solution containing 20 mM unlabeled Z_IGF1R:4551_-GGGC. Cells in another three dishes were saturated with 500 μL blocking solution containing 20 mM unlabeled IGF-1. To the remaining three Petri dishes, 500 μL complete RPMI medium was added in order to obtain the same volume. Five minutes later, 500 μL of a solution containing 5 nM of ^125^I-IGF-1 was added to each dish, followed by incubation during 1.5 h at 37 °C under 5 % CO_2_. After the incubation, the media were collected, the cells were washed with incomplete medium, 500 μL trypsin–EDTA solution was added, and the cells were incubated for 10 min. The detached cells were diluted in 500 μL complete media, resuspended, and transferred to the appropriate fraction tubes. The radioactivity of media and cells was measured using an automated gamma counter and the percent of cell-bound radioactivity was calculated. In the second set of experiments, saturation of ^99m^Tc-Z_IGF1R:4551_-GGGC was tested. The protocol was similar to the previous one, but the concentration of blocking solutions was 5 mM.

For internalization studies, cells (750,000 per dish) were incubated with the labeled compound (1.5 nM) at 37 °C, 5 % CO_2_. At predetermined time points (1, 2, 4, 8, and 24 h after incubation start), the medium from a set of 3 dishes was removed. To collect the membrane-bound radioactivity, the cells were treated with 0.2 M glycine buffer containing 4 M urea, pH 2.5, for 5 min on ice, and the acid fraction was collected. Cells containing internalized radioactivity were detached by treatment with 1 M NaOH at 37 °C for 0.5 h and collected. The percentage of membrane-bound and internalized radioactivity was calculated for each time point.

The affinity was measured using a LigandTracer Instrument (Ridgeview Instruments AB) at room temperature, as previously described (Björkelund et al. 2011). Briefly, a Petri dish (Nunclon, diameter 100 mm, containing 3 mL culture medium) with DU-145 cells was attached to the rotating table of the instrument. After 10 min baseline run, ^99m^Tc-Z_IGF1R:4551_-GGGC was added to the medium to obtain a ligand concentration of 0.3 nM, and the uptake curve was recorded for 150 min. Thereafter, the ligand concentration was increased to 0.9 nM, and the uptake curve was recorded for another 140 min. The final uptake measurement was performed with ^99m^Tc-Z_IGF1R:4551_-GGGC 1.8 nM concentration for 140 min. Then the ^99m^Tc-Z_IGF1R:4551_-GGGC-containing medium was aspirated, 3 mL of fresh medium was added, and the dissociation curve was measured. Interaction analysis and calculation of equilibrium dissociation constant (*K*_D_) were performed with TracerDrawer software (Ridgeview Instruments AB). Four additional measurements were performed at 4 °C to exclude the influence of ligand internalization.

### In vivo studies

The animal study was planned and performed in accordance with national legislation on protection of laboratory animals and was approved by the Ethics Committee for Animal Research in Uppsala.

For tumor grafting, 5 × 10^6^ prostate cancer DU-145 cells (in Matrigel, BD Biosciences) were subcutaneously implanted in the right hind leg of male Balb/c nu/nu mice. Xenografts were allowed to grow for 2 weeks. At the time of experiment, the animal weight was 21.5 ± 1.6 g, and the tumor weight was 290 ± 170 mg. Before implantation of the breast cancer MCF-7 cells, female Balb/c nu/nu mice were pre-implanted with estradiol pellets (0.5 mg/d, 21 days; Innovative Research of America). The cells, 5 × 10^6^ in Matrigel, were implanted in the right hind leg 2 weeks before experiment. At the time of experiment, the animal weight was 20.5 ± 0.8 g, and the tumor weight was 60 ± 30 mg. Immediately before experiments, the tumor-bearing mice were randomized in groups containing four animals each.

The animals (four mice per data point) were injected intravenously with 60 kBq of radiolabeled conjugate per mouse in 100 μL PBS. The protein dose was adjusted to 1 µg per mouse by addition of non-labeled Z_IGF1R:4551_-GGGC. The mice were sacrificed at predetermined time points by an intraperitoneal injection of anesthesia, Ketalar–Rompun solution (20 μL/g body weight; Ketalar, 10 mg/mL; Rompun, 1 mg/mL). Blood, lung, liver, spleen, stomach, kidney, salivary gland, tumor, samples of colon, pancreas, muscle, bone, as well as the rest of intestines with their content were collected. Organs and tissue samples were weighed, and their radioactivity was measured. The tissue uptake values were calculated as percent of injected activity per gram tissue (%IA/g).

To evaluate in vivo kinetics, the biodistribution of ^99m^Tc-Z_IGF1R:4551_-GGGC was measured in mice bearing DU-145 xenografts at 1, 4, and 8 h after injection. To verify specificity of in vivo targeting, the effect of in vivo receptor saturation was tested. For this purpose, one group of mice was injected in 60 kBq of ^99m^Tc-Z_IGF1R:4551_-GGGC, the protein dose being adjusted to 40 µg per mouse using non-labeled Z_IGF1R:4551_-GGGC. This group was euthanized at 4 h after injection. For direct comparison, another group of tumor-bearing mice was injected with 60 kBq (1 µg) of [^99m^Tc(CO)_3_]^+^-(HE)_3_-Z_IGF1R:4551_, and the biodistribution was measured at 4 h after injection.

In addition, the biodistribution of ^99m^Tc-Z_IGF1R:4551_-GGGC and [^99m^Tc(CO)_3_]^+^-(HE)_3_-Z_IGF1R:4551_ was compared at 4 h after injection of 60 kBq (1 µg) in female Balb/c nu/nu mice bearing MCG-7 breast cancer xenografts. The goal was to confirm the in vivo data using different types of tumors (breast vs prostate cancer) as well as possible differences attributed to sex (female vs male).

To obtain a visual confirmation of biodistribution data, one mouse with a DU-145 xenograft and one mouse with a MCF-7 xenograft were injected with ^99m^Tc-Z_IGF1R:4551_-GGGC (1 MBq, 1 μg, in 100 μL PBS) and imaging was performed 4 h later. Immediately before imaging, the animals were killed by an overdose of anesthetics. Imaging was performed using a GE Infinia gamma camera equipped with a low-energy high-resolution collimator. Static images (30 min) were obtained with a zoom factor of 2 in a 256 × 256 matrix. The images were evaluated using Osiris 4.19 software (University Hospital of Geneva, Switzerland). In each animal, a region of interest was drawn around the tumor. The same region was copied to the contralateral thigh. Tumor-to-contralateral thigh ratios were calculated based on average counts in the regions of interest.

## Results

### Production and labeling

**Fig. 1:**
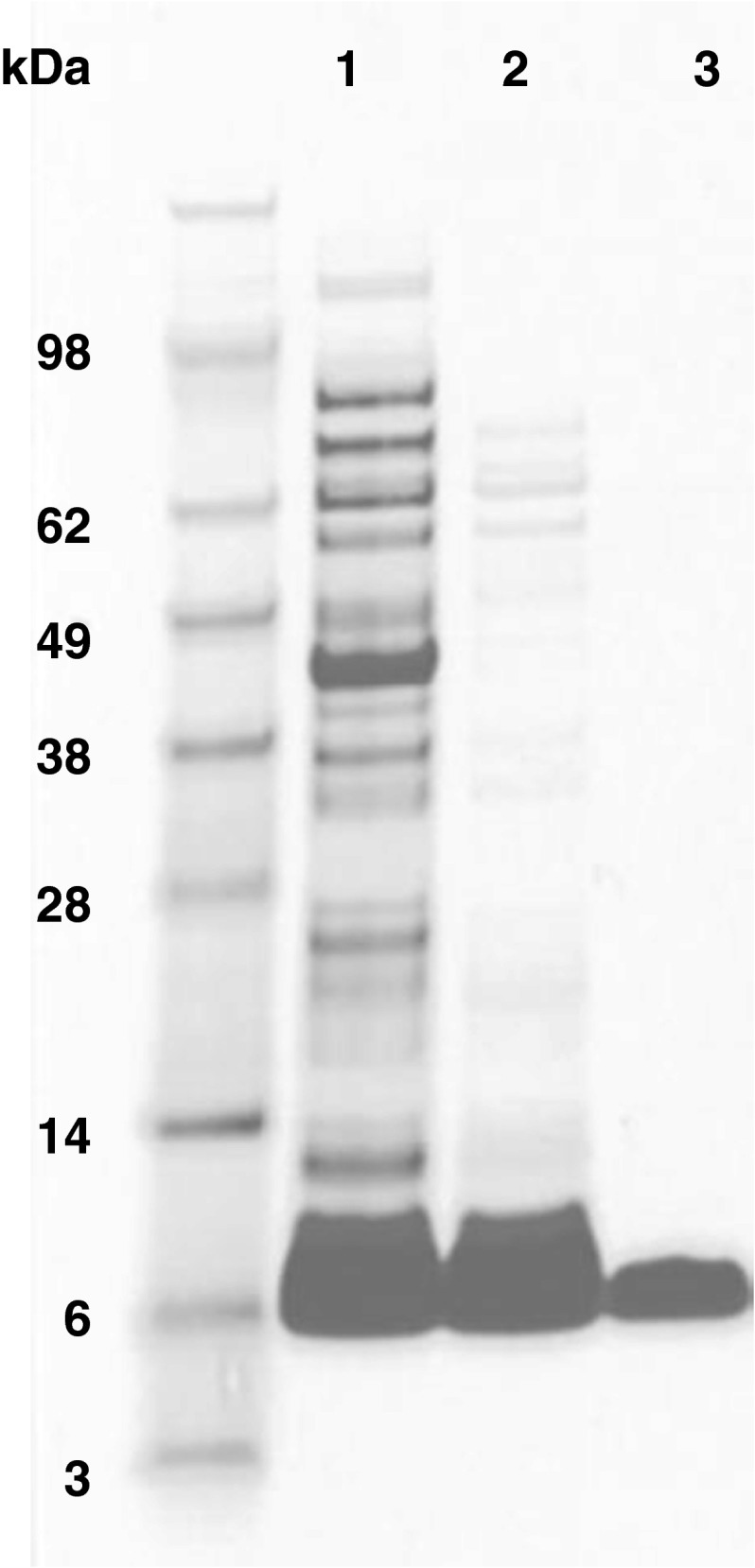
SDS-PAGE analysis of samples taken during the purification of Z_IGF1R:4551_-GGGC. *Lane 1* sample after cell lysis and initial clarification, *lane 2* sample after heat treatment, *lane 3* sample after HPLC purification

The IGF-1R targeting affibody molecule, Z_IGF1R:4551_-GGGC, was expressed in *Escherichia coli* and purified by heat treatment followed by reversed-phase high-performance liquid chromatography (Fig. 1). The ESI MS confirmed authenticity of Z_IGF1R:4551_-GGGC as the deconvoluted mass (6297.0 Da) was in an excellent agreement with the theoretical molecular mass of 6297.0 Da. According to HPLC analysis, a purity of Z_IGF1R:4551_-GGGC was more than 99.5 %.

**Fig. 2:**
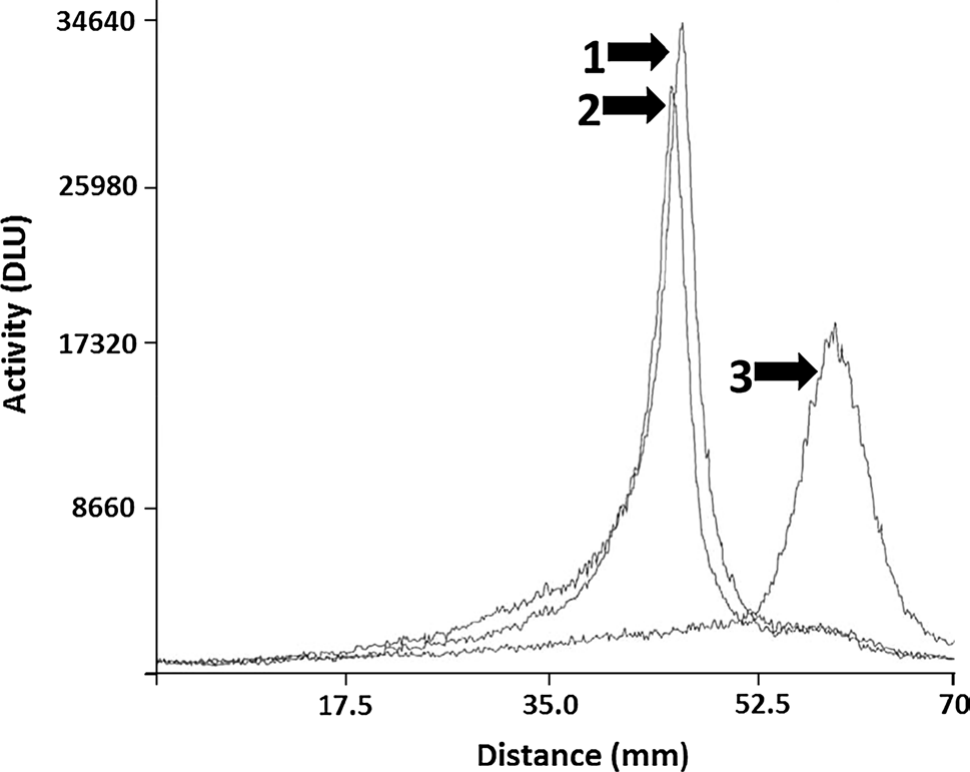
SDS-PAGE analysis of ^99m^Tc-Z_IGF1R:4551_-GGGC stability in serum. Distribution of radioactivity along lanes was visualized using Cyclone™ Storage Phosphor System. The signal was measured as digital light units (DLU) and was proportional to the radioactivity in given point of the SDS-PAGE gel. *1* ^99m^Tc-Z_IGF1R:4551_-GGGC sample, which was incubated in murine serum at 37 °C for 1 h. *2* ^99m^Tc-Z_IGF1R:4551_-GGGC sample, which was incubated in PBS at 37 °C for 1 h. *3* ^99m^Tc-pertechnetate was used as a marker for low molecular weight compounds

Z_IGF1R:4551_-GGGC was successfully labeled with ^99m^Tc, with a resulting yield of 96.9 ± 2.9 %. The purification was performed using disposable size-exclusion NAP-5 columns, and the resulting radiochemical purity was 99.8 ± 0.4 %. A serum stability test revealed very good serum stability after 1 h incubation at 37 °C. The ITLC results showed 97.2 ± 1.7 % stability, while the SDS-PAGE analysis showed no measurable release of radionuclide from samples incubated in serum (see Fig. 2). The only observed radioactivity peaks corresponded to the migration path of the Z_IGF1R:4551_-GGGC affibody molecule. There was no observed aggregation of affibody molecules or transchelation of radionuclide to blood plasma proteins. Similarly, the stability in PBS was also high as shown in Fig. 2.

### In vitro characterization

**Fig. 3:**
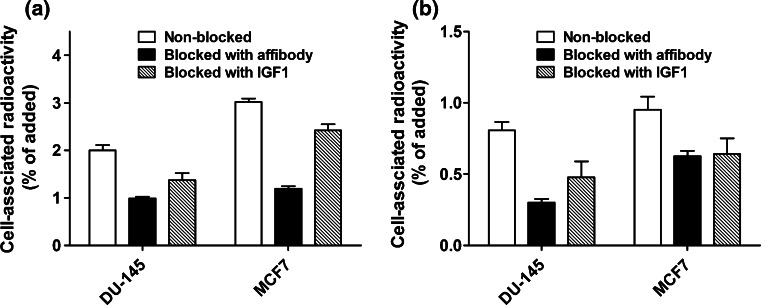
In vitro specificity test of Z_IGF1R:4551_-GGGC binding to IGF-1R on DU-145 (human prostate cancer) and MCF-7 (human breast cancer) cell lines. **a** Pre-saturation of receptors with unlabeled Z_IGF1R:4551_ or IGF-1 caused significant (*p* < 0.05 in Student’s *t* test) reduction of cell-bound ^99m^Tc-Z_IGF1R:4551_-GGGC radioactivity, **b** pre-saturation of receptors with unlabeled Z_IGF1R:4551_ or IGF-1 caused significant (*p* < 0.05 in Student’s *t* test) reduction of cell-bound ^125^I-IGF radioactivity

The results of in vitro binding specificity test are presented in Fig. 3. A pre-saturation of receptors by adding a large molar excess of unlabeled affibody molecules or a natural IGF-1R ligand, IGF-1, caused significant (*p* < 0.05) reduction of ^99m^Tc-Z_IGF1R:4551_-GGGC uptake in IGF-1R-expressing DU-145 and MCF-7 cells (Fig. 3a). In addition, a large molar excess of non-labeled Z_IGF1R:4551_-GGGC reduced significantly binding of radiolabeled natural ligand, ^125^I-IGF-1, to these cell lines (Fig. 3b). This demonstrated that ^99m^Tc-Z_IGF1R:4551_-GGGC could bind specifically to IGF1R and suggested that the affibody molecule has the same or an overlapping binding site as the natural ligand.

**Fig. 4:**
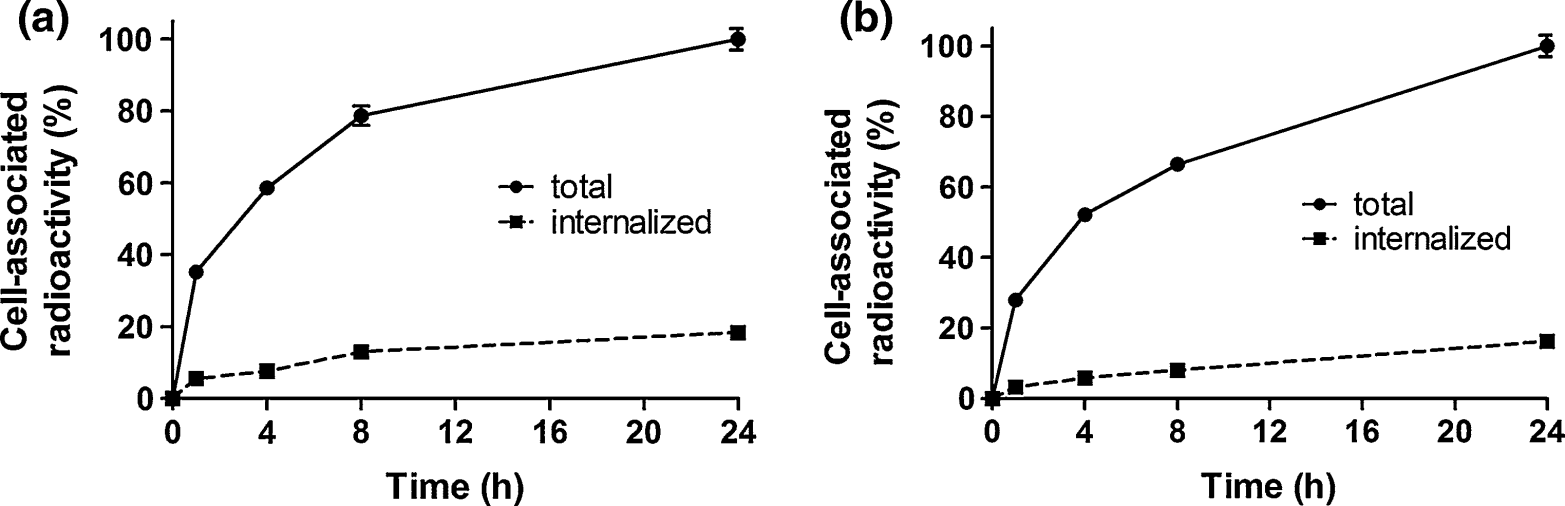
Binding and internalization of ^99m^Tc-Z_IGF1R:4551_-GGGC at 37 °C by prostate cancer DU-145 (**a**) and breast cancer MCF-7 (**b**) cells. Data are normalized to a maximum cell-bound radioactivity and presented as average value from 3 cell dishes ± SD. *Error bars* might not be seen because they are smaller than *point symbols*

**Fig. 5:**
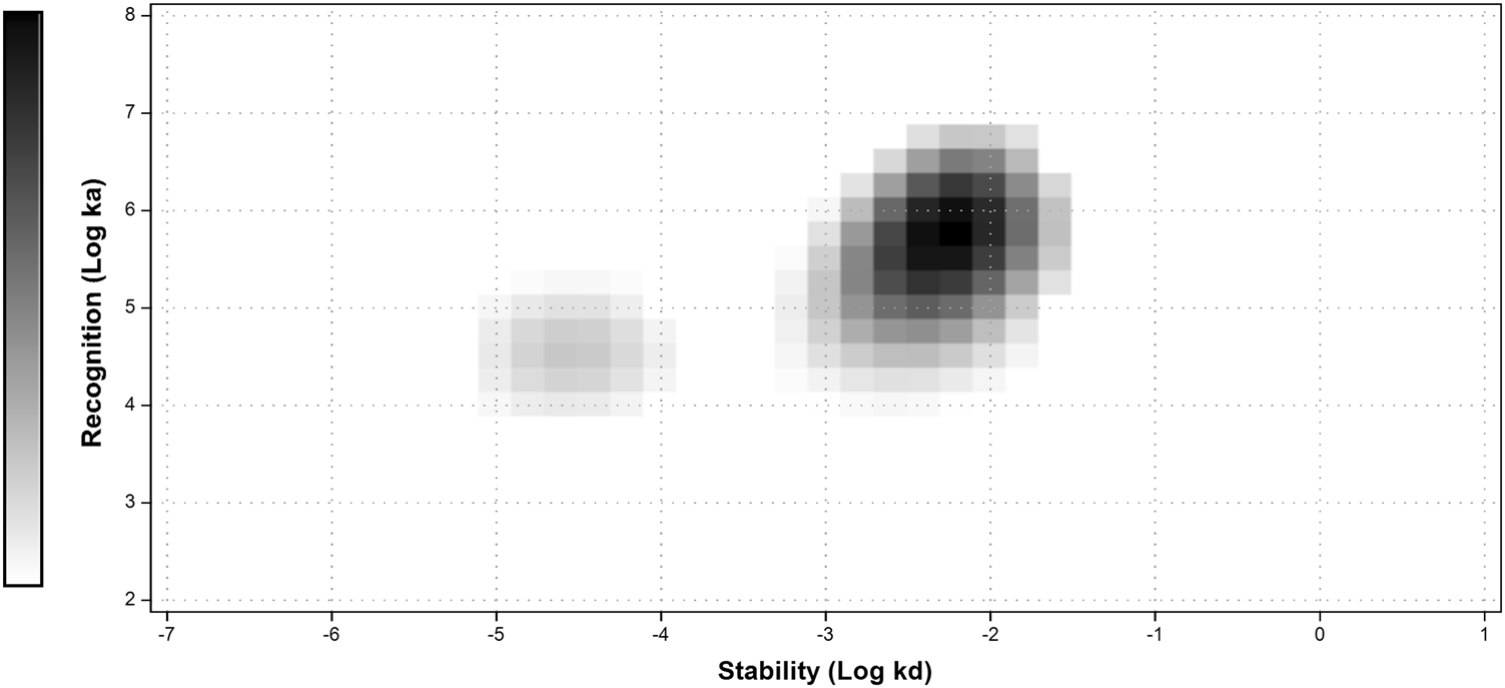
Interaction map: interaction between ^99m^Tc-Z_IGF1R:4551_-GGGC and DU-145 cells. The two simultaneous interactions observed as distinct processes in the map. The *grey scale* reflects contribution of a process, where *darker areas* represent larger contributions

Data concerning internalization of ^99m^Tc-Z_IGF1R:4551_-GGGC by DU-145 and MCF-7 cells are presented in Fig. 4a and b. The internalization pattern by both cell lines was similar, showing a relatively low fraction of internalized radioactivity. Although a gradual increase of the internalized fraction was observed for both cell lines, only 18.3 ± 0.8 % of the total cell-associated radioactivity were internalized at 24 h after incubation start for DU-145 and 16.2 ± 0.6 % for MCF-7 cells. The binding affinity of ^99m^Tc-Z_IGF1R:4551_-GGGC to DU-145 cells was also measured. Similar to a previous study (Orlova et al. 2013), the interaction did not follow a 1:1 Langmuir adsorption model and was found to be a combination of two independent processes when subjected to an Interaction Map analysis (Fig. 5). The Interaction Map shows the two processes where the higher affinity interaction is 844 pM and the lower affinity interaction is 12 nM.

### Biodistribution in tumor-bearing mice

**Fig. 6:**
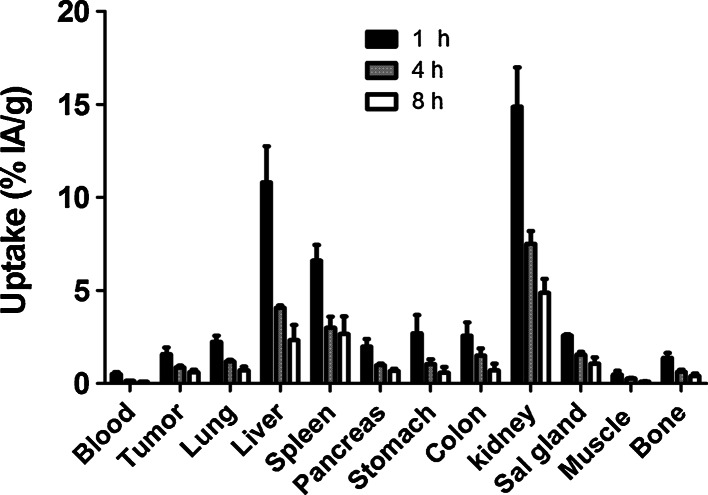
Biodistribution of ^99m^Tc-Z_IGF1R:4551_-GGGC in Balb/c nu/nu mice with subcutaneous DU-145 prostate cancer xenografts as a function of time. Data are presented as an average %IA/g and standard deviation for four mice

**Fig. 7:**
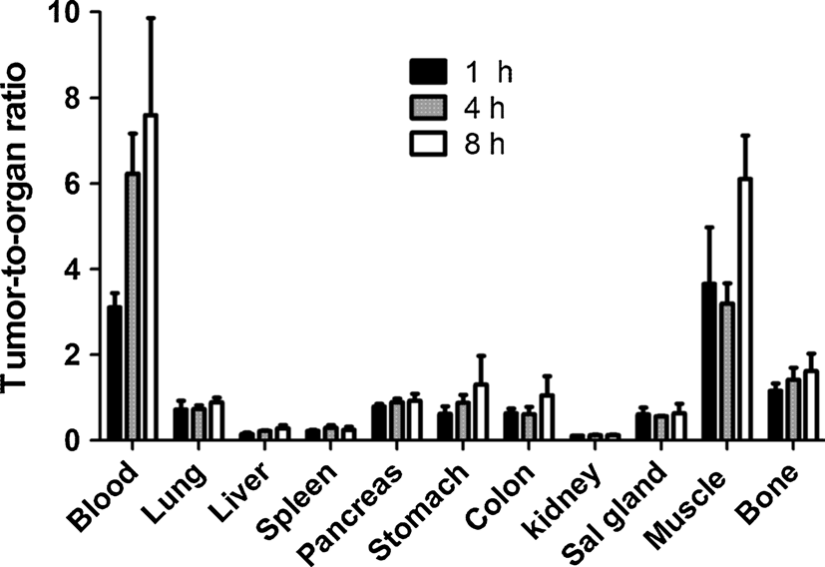
Tumor-to-organ ratios of ^99m^Tc-Z_IGF1R:4551_-GGGC in Balb/c nu/nu mice with subcutaneous DU-145 prostate cancer xenografts. Data are presented as an average value and standard deviation for four mice

Data concerning biodistribution of ^99m^Tc-Z_IGF1R:4551_-GGGC in Balb/c nu/nu mice bearing DU-145 prostate cancer xenografts (injected dose 1 µg) as a function of time are present in Table 1 and Figs. 6 and 7.

**Table 1:** Specificity of targeting of IGF-1R in vivo

	1 µg	40 µg
Blood	0.14 ± 0.01*	0.22 ± 0.04
Lung	1.19 ± 0.06*	0.61 ± 0.05
Liver	4.08 ± 0.1	2.85 ± 1.3
Spleen	3 ± 0.6	3.13 ± 2.4
Pancreas	0.98 ± 0.1*	0.33 ± 0.06
Stomach	1.04 ± 0.3*	0.48 ± 0.05
Colon	1.5 ± 0.4*	0.7 ± 0.4
Kidney	7.5 ± 0.7	6.4 ± 0.5
Salivary gland	1.6 ± 0.1*	1.0 ± 0.1
Tumor	0.9 ± 0.1*	0.5 ± 0.1
Muscle	0.27 ± 0.01	0.26 ± 0.1
Bone	0.63 ± 0.1	0.43 ± 0.1

Biodistribution (4 h after injection) of ^99m^Tc-Z_IGF1R:4551_-GGGC in Balb/c nu/nu mice bearing IGR-1R-expressing DU-145 prostate cancer xenografts injected with 1 or 40 μg protein. Uptake of ^99m^Tc-Z_IGF1R:4551_-GGGC in tumors and IGF-1R-expressing organs (lung, pancreas, stomach, and colon) was significantly lower in animals injected with higher dose of Z_IGF1R:4551_-GGGC

The data are expressed as %IA/g and represent the average value from 4 animals ± standard deviation

* Significant difference (*p* < 0.05) in uptake between mice injected with 1 and 40 μg protein

The data in Table 1 show that the tumor uptake at 4 h after injection of 40 µg of ^99m^Tc-Z_IGF1R:4551_-GGGC (0.5 ± 0.1 %IA/g) was significantly (*p* < 0.05) lower than the uptake after injection of 1 µg (0.9 ± 0.1 %IA/g). Moreover, there was a significantly lower uptake in IGF-1R-expressing tissues, such as lung, pancreas, stomach, colon, and salivary glands after injection of saturating amount of unlabeled Z_IGF1R:4551_-GGGC together with ^99m^Tc-Z_IGF1R:4551_-GGGC. This demonstrates the saturable character of the binding of tracer to IGF-1R also in vivo and suggests its specificity.

The biodistribution of the conjugate was characterized by rapid clearance from blood and normal tissues (Fig. 6). A low accumulation of radioactivity in the intestines with their content (3.7 ± 0.6 %IA at 1 h after injection and 3.2 ± 1.3 %IA at 4 h) indicated that hepatobiliary excretion played a minor role in the clearance. The clearance was, most likely, predominantly renal. However, the retention of radioactivity in kidneys was low with a rapid decrease between time points (15 ± 2, 7.5 ± 0.7 and 4.9 ± 0.8 %IA/g at 1, 4, and 8 h after injection, respectively), indicating a non-residualizing character of the label. The high initial uptake of radioactivity in the liver (11 ± 2 %IA/g) was followed by an appreciable decrease reaching 4.1 ± 0.1 %IA/g at 4 h after injection and 2.33 ± 0.8 %IA/g at 8 h. The release of radioactivity from the tumor (1.8-fold decrease at 4 h after injection compared to 1 h) was slower than the clearance from blood (3.6-fold between these time points) and release from liver (2.7-fold).

Tumor-to-organ ratios are presented in Fig. 7. There was a significant (*p* < 0.05) increase of tumor-to-blood and tumor-to-liver ratios between 1 and 4 h after injection but no significant increase in tumor-to-organ ratios between 4 and 8 h after injection. This indicates that 4 h after injection could be an optimal imaging time.

The imaging potential of ^99m^Tc-Z_IGF1R:4551_-GGGC was further investigated by comparing its biodistribution with the biodistribution of the previously developed IGF-1R-interacting probe [^99m^Tc(CO)_3_]^+^-(HE)_3_-Z_IGF1R:4551_. Data concerning the comparative biodistribution in mice bearing DU-145 and MCF-7 xenografts are present in Table 2. The comparison of tumor-to-organ ratios is shown in Table 3. The influence of labeling approach on biodistribution was very similar in both models. Noticeably, there was a significantly (*p* < 0.001) faster blood clearance for ^99m^Tc-Z_IGF1R:4551_-GGGC in both male and female mice. The accumulation of radioactivity in the kidneys was appreciably different for the two compounds, where ^99m^Tc-Z_IGF1R:4551_-GGGC was found to give a 15- to 17-fold lower renal uptake compared to [^99m^Tc(CO)_3_]^+^-(HE)_3_-Z_IGF1R:4551_. Uptake of [^99m^Tc(CO)_3_]^+^-(HE)_3_-Z_IGF1R:4551_ in liver and spleen was lower in both cases. The higher liver uptake of ^99m^Tc-Z_IGF1R:4551_-GGGC was not associated with a high hepatobiliary excretion. The levels of radioactivity in the intestines with content for ^99m^Tc-Z_IGF1R:4551_-GGGC reached 3.15 ± 1 %IA and 3.14 ± 0.25 %IA for male and female mice, respectively, while the corresponding values for [^99m^Tc(CO)_3_]^+^-(HE)_3_-Z_IGF1R:4551_ were 6.41 ± 0.4 %IA/g and 4.68 ± 1 %IA/g. The tumor uptake was similar for both compounds in mice bearing MCF-7 xenografts, but the tumor uptake of ^99m^Tc-Z_IGF1R:4551_-GGGC in DU-145 xenografts was significantly lower than the uptake of [^99m^Tc(CO)_3_]^+^-(HE)_3_-Z_IGF1R:4551_.

**Table 2:** Comparative biodistribution of ^99m^Tc-Z_IGF1R:4551_-GGGC and [^99m^Tc(CO)_3_]^+^-(HE)_3_-Z_IGF1R:4551_ in mice bearing DU-145 and MCF-7 xenografts at 4 h after injection

	DU-145		MCF-7	
	^99m^Tc-Z_IGF1R:4551_-GGGC	[^99m^Tc(CO)_3_]^+^-(HE)_3_-Z_IGF1R:4551_	^99m^Tc-Z_IGF1R:4551_-GGGC	[^99m^Tc(CO)_3_]^+^-(HE)_3_-Z_IGF1R:4551_
Blood	0.14 ± 0.01*	0.47 ± 0.03	0.2 ± 0.07*	0.36 ± 0.04
Lung	1.19 ± 0.07*	2.02 ± 0.5	1.6 ± 0.3	1.6 ± 0.2
Liver	4.08 ± 0.1*	3.5 ± 0.4	6.07 ± 0.1*	3.06 ± 0.4
Spleen	3.0 ± 0.6*	1.56 ± 0.2	3.11 ± 0.7*	1.43 ± 0.3
Pancreas	0.98 ± 0.1	1.13 ± 0.1	1.41 ± 0.2	1.1 ± 0.15
Stomach	1.04 ± 0.3	1.38 ± 0.2	1.44 ± 0.3	1.19 ± 0.2
Colon	1.5 ± 0.4	1.6 ± 0.2	1.4 ± 0.1	1.55 ± 0.2
Kidney	7.5 ± 0.7*	116 ± 7	9.5 ± 0.4*	164 ± 3
Sal gland	1.6 ± 0.1	1.9 ± 0.3	1.4 ± 0.2	1.2 ± 0.2
Tumor	0.9 ± 0.1*	1.3 ± 0.2	1.2 ± 0.2	1.6 ± 0.3
Muscle	0.27 ± 0.02	0.31 ± 0.06	0.29 ± 0.07	0.26 ± 0.07
Bone	0.63 ± 0.1	0.61 ± 0.15	0.59 ± 0.06	0.6 ± 0.1

The data are expressed as %IA/g and present the average value from 4 animals ± standard deviation

* Significant difference (*p* < 0.05) between the uptake of ^99m^Tc-Z_IGF1R:4551_-GGGC and [^99m^Tc(CO)_3_]^+^-(HE)_3_-Z_IGF1R:4551_ Affibody molecules in mice bearing DU-145 and MCF-7 xenografts

**Table 3:** Comparison of tumor-to-organ ratios for ^99m^Tc-Z_IGF1R:4551_-GGGC and [^99m^Tc(CO)_3_]^+^-(HE)_3_-Z_IGF1R:4551_ at 4 h after injection in mice bearing DU-145 and MCF-7 xenografts

	DU-145		MCF-7	
	^99m^Tc-Z_IGF1R:4551_-GGGC	[^99m^Tc(CO)_3_]^+^-(HE)_3_-Z_IGF1R:4551_	^99m^Tc-Z_IGF1R:4551_-GGGC	[^99m^Tc(CO)_3_]^+^-(HE)_3_-Z_IGF1R:4551_
Blood	6.2 ± 0.9*	2.7 ± 0.3	6.9 ± 1.0*	4.48 ± 0.9
Lung	0.7 ± 0.1	0.7 ± 0.1	0.8 ± 0.1	0.9 ± 0.3
Liver	0.21 ± 0.02*	0.37 ± 0.05	0.20 ± 0.02*	0.5 ± 0.1
Spleen	0.3 ± 0.05*	0.8 ± 0.1	0.4 ± 0.1*	1.1 ± 0.2
Pancreas	0.9 ± 0.1*	1.13 ± 0.07	0.9 ± 0.2*	1.5 ± 0.3
Stomach	0.9 ± 0.2	0.93 ± 0.05	0.88 ± 0.05*	1.34 ± 0.3
Colon	0.6 ± 0.2	0.8 ± 0.1	0.9 ± 0.2	1.0 ± 0.2
Kidney	0.12 ± 0.01*	0.01 ± 0.001	0.13 ± 0.02*	0.01 ± 0.003
Sal gland	0.56 ± 0.01*	0.66 ± 0.07	0.91 ± 0.2	1.3 ± 0.3
Muscle	3.2 ± 0.5*	4.14 ± 0.5	4.55 ± 1.8	6.38 ± 1.65
Bone	1.4 ± 0.3*	2.2 ± 0.3	2.1 ± 0.3*	2.6 ± 0.2

The data presented are average ratios together with standard deviations in four mice

* Significant difference (*p* < 0.05) between the tumor-to-organ ratios of ^99m^Tc-Z_IGF1R:4551_-GGGC and [^99m^Tc(CO)_3_]^+^-(HE)_3_-Z_IGF1R:4551_ Affibody molecules

Tumor-to-blood ratios were higher for ^99m^Tc-Z_IGF1R:4551_-GGGC in both models (Table 3), while tumor-to-liver, tumor-to-spleen, and tumor-to-pancreas ratios were significantly higher for [^99m^Tc(CO)_3_]^+^-(HE)_3_-Z_IGF1R:4551_.

**Fig. 8:**
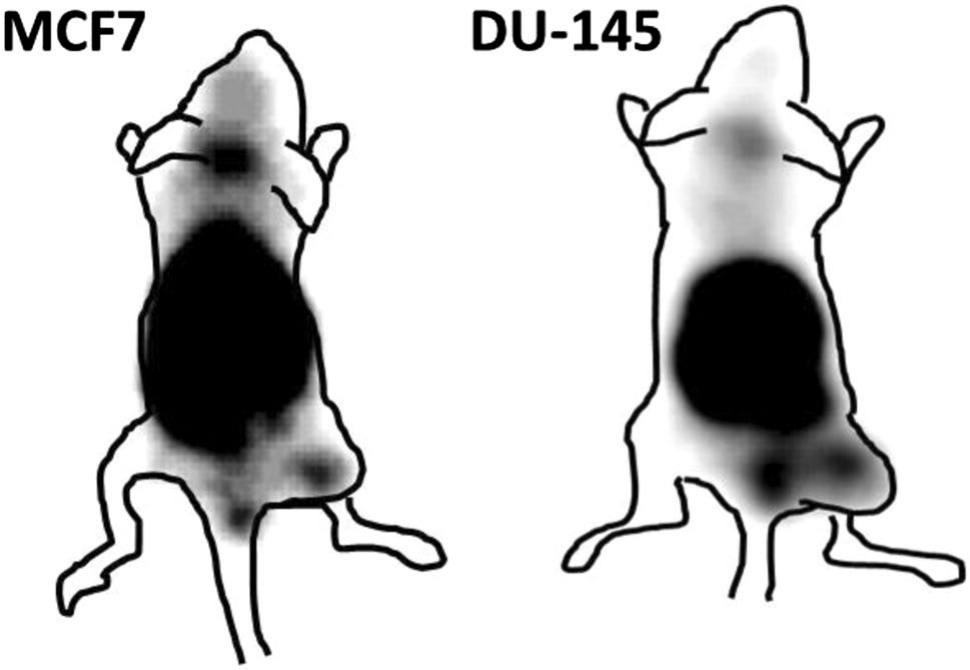
Imaging of IGF-1R expression in DU-145 prostate cancer and MCF7 breast cancer xenografts in NMRI nu/nu mice using ^99m^Tc-Z_IGF1R:4551_-GGGC. Planar γ-camera images were acquired at 4 h after injection

Gamma camera imaging showed that ^99m^Tc-Z_IGF1R:4551_-GGGC was capable of visualizing subcutaneous IGF-1R-expressing DU-145 and MCF-7 xenografts (Fig. 8). From this image, the tumor-to-contralateral site ratio was determined to 3.3 for MCF-7 and 3.9 for DU-145. The image also indicated the feasibility of detecting IGF-1R-expressing lung metastases. At the same time, radioactivity accumulation in salivary glands, kidneys, liver, and stomach was on the same level or higher than in tumors.

## Discussion

Determination of IGF-1R expression level in tumors may yield information that can help predict response to different treatment modalities as well as clinical outcome for the patient. Toward this goal, we have in this study investigated a new probe, ^99m^Tc-Z_IGF1R:4551_-GGGC, for determining the level of IGF-1R expression by radionuclide molecular imaging in pre-clinical models of prostate and breast cancer. The labeling of Z_IGF1R:4551_-GGGC with ^99m^Tc was successful, with yields exceeding 96 %. The resulting compound was characterized by an excellent stability with no signs of radionuclide release, aggregation, or a radionuclide transchelation to plasma proteins (Fig. 2). These results are consistent with previous studies where affibody molecules were fitted with a GGGC peptide for ^99m^Tc-labeling, which showed that C-terminal positioning of the cysteine-containing chelators provides high stability with a low release of ^99m^Tc-pertechnetate in serum due to a favorable geometry of the chelate (Tran et al. 2007; Wållberg et al. 2011). The binding specificity test indicated that the binding of ^99m^Tc-Z_IGF1R:4551_-GGGC to IGF-1R-expressing cell lines (human prostate cancer cells DU-145 and human breast cancer cells MCF-7) was receptor mediated (Fig. 3), suggesting that labeling has not influenced the ability of Z_IGF1R:4551_-GGGC to bind specifically to IGF-1R. The presence of two distinct interaction processes as shown by the interaction map (Fig. 5)—one with higher affinity (844 pM) and one with lower affinity (12 nM)—is consistent with previous data obtained for [^99m^Tc(CO)_3_]^+^-(HE)_3_-Z_IGF1R:4551_ suggesting that Z_IGF1R:4551_ binds to two binding sites on IGF-1R (Orlova et al. 2013). Since Z_IGF1R:4551_ is competing with IGF-1 for binding to the receptor (Li et al. 2010), a possible explanation for the two-process interaction may be that it binds to the receptor similarly as IGF-1, which has been found to interact at two positions on IGF-1R with negative cooperativity (Surinya et al. 2008). The internalization rate was relatively low for both DU-145 and MCF-7 cell lines, with less than 20 % of internalized activity after 24 h (Fig. 4). This suggested that the use of non-residualizing labels is feasible.

The in vivo receptor saturation experiment (Table 1) demonstrated a significantly lower uptake of ^99m^Tc-Z_IGF1R:4551_-GGGC in DU-145 xenografts after injection of a saturating dose of Z_IGF1R:4551_-GGGC. Together with the results of the in vitro specificity test, this suggests that the tumor uptake in vivo was receptor mediated. The uptake in lung, pancreas, stomach, colon, and salivary glands (i.e., tissues expressing IGF-1R) was also lower after injection of the saturating protein dose. Furthermore, uptake of affibody molecules targeting other receptors, e.g., HER2 or PDGFRβ, was 2- to 4-fold lower in these organs (Ahlgren et al. 2009; Wållberg et al. 2011; Tolmachev et al. 2014) than uptake of ^99m^Tc-Z_IGF1R:4551_-GGGC when 1 µg was injected. These affibody molecules have the same scaffold but different amino acids involved in binding and might be considered as negative not-IGF-1R-binding controls for Z_IGF1R:4551_-GGGC. Uptake saturability and its significantly higher level in comparison with uptake of not-IGF-1R-binding counterparts indicate that the ^99m^Tc-Z_IGF1R:4551_-GGGC accumulation in these organs was also receptor mediated. The specific uptake in IGF-1R-expressing organs suggests that the murine model is adequate for in vivo studies concerning IGF-1R targeting, which is essential for translation of murine data to humans.

The biodistribution of ^99m^Tc-Z_IGF1R:4551_-GGGC was characterized by fast blood clearance, which is important for high-sensitivity molecular imaging. The maximum tumor-to-blood ratios for ^99m^Tc-Z_IGF1R:4551_-GGGC were obtained already at 4 h after injection (Fig. 7). The tumor-to-blood ratios provided by Z_IGF1R:4551_-GGGC at this time point (6.2 ± 0.9 and 6.9 ± 1.03, for DU-145 and MCF-7, respectively) (Table 3) were on the same level or better than ^111^In-labeled (Fab′)_2_-fragments of anti-IGF-1R antibody R1507 at 24 h after injection (7.5 ± 1.4 and 2.4 ± 0.4 in SUM149 and EW-5 xenografts, respectively) (Heskamp et al. 2012; Fleuren et al. 2013). The tumor-to-blood ratios were also higher than the ones previously reported for anti-IGF-1R antibody R1507 (2.8 ± 0.7) at 72 h after injection (Heskamp et al. 2012). Of course, such comparison should be taken very cautiously for two reasons. First, the models were different, which can be associated with different levels of IGF-1R expression, as well as with different degree of vascularization and even vascular permeability. Second, R1507 antibody does not cross react with murine IGF-1R (Heskamp et al. 2012), which creates an idealized condition for this antibody and its derivatives, excluding interaction with IGF-1R in normal tissues.

Previous studies, that were performed using anti-HER2 affibody molecules, suggest that the use of a GGGC chelator for ^99m^Tc at the C-terminus provides a non-residualizing label (Wållberg et al. 2011). Because of the slow internalization of affibody molecules by cancer cells, this has a small effect on tumor uptake, but enables a substantial reduction of radioactivity retention in excretory organs (kidneys and liver), where internalization is rapid. On the opposite, the use of the peptide chelator HEHEHE at the N-terminus provides a residualizing label for [^99m^Tc(CO)_3_]^+^ (Tolmachev et al. 2010b). In this case, the retention of radionuclides in excretory organs is rather high, but the liver uptake is low, which is associated with the presence of negatively charged and hydrophilic amino acids (Hofström et al. 2013). Overall, this provided an approximately equal uptake of anti-HER2 affibody molecules in liver (0.9 ± 0.1 and 0.88 ± 0.05 %IA/g at 4 h after injection, for ^99m^Tc-GGGC and [^99m^Tc(CO)_3_]^+^-(HE)_3_, respectively), but much lower renal retention of technetium-99m in the case of GGGC chelator (8.2 ± 2.9 and 70 ± 10 %IA/g at 4 h after injection, for ^99m^Tc-GGGC and [^99m^Tc(CO)_3_]^+^-(HE)_3_, respectively). In this study, we confirmed that the clearance of ^99m^Tc-Z_IGF1R:4551_-GGGC from kidney was rapid (Fig. 6), and renal radioactivity concentration was 15- to 17-fold lower than the concentration of [^99m^Tc(CO)_3_]^+^-(HE)_3_-Z_IGF1R:4551_ at 4 h after injection (Table 2). However, the liver uptake of ^99m^Tc-Z_IGF1R:4551_-GGGC was significantly higher than the uptake of [^99m^Tc(CO)_3_]^+^-(HE)_3_-Z_IGF1R:4551_ at this time point (Table 2). Per se, clearance of radioactivity from liver was rapid (11 ± 2 vs 4.1 ± 0.1 %IA/g at 1 and 4 h, respectively) in the case ^99m^Tc-Z_IGF1R:4551_-GGGC (Fig. 6). This was different from [^99m^Tc(CO)_3_]^+^-(HE)_3_-Z_IGF1R:4551_, where reduction of hepatic activity between 1 h (2.3 ± 0.2 %IA/g) and 4 h after injection (1.7 ± 0.3 %IA/g) was minor (Orlova et al. 2013). We can state that the effect of chelators on the hepatic retention of radioactivity was similar for anti-HER2 and anti-IGF-1R affibody molecules, but the initial hepatic uptake of ^99m^Tc-Z_IGF1R:4551_-GGGC was much higher. It is important to note that the hepatic uptake of radiolabeled Z_IGF1R:4551_ was not saturable neither in this, nor in previous studies using this construct, irrespective of the labels used (Tolmachev et al. 2012a; Orlova et al. 2013). Thus, hepatic uptake of ^99m^Tc-Z_IGF1R:4551_-GGGC is, most likely, not-IGF-1R specific. Furthermore, hepatic uptake was not associated with the affibody scaffold, as the hepatic uptake of the anti-HER2 ^99m^Tc-Z_HER2:V2_-GGGC affibody molecule with the same scaffold as Z_IGF1R:4551_ was much lower (Wållberg et al. 2011). Therefore, the major difference in hepatic uptake between anti-HER2 and anti-IGF-1R affibody molecules is most likely a consequence of differences in their binding sites.

**Fig. 9:**
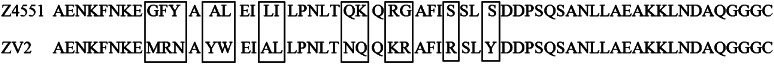
Sequence comparison between the anti-IGF-1R affibody molecule Z_IGF1R:4551_-GGGC and anti-HER2-affibody molecule Z_HER2:V2_-GGGC. Amino acids in the binding site are *boxed*

The binding site of Z_IGF1R:4551_ contains two glycines, two leucines, two serines, and one of each: phenylalanine, tyrosine, alanine, isoleucine, glutamine, lysine, and arginine (Fig. 9). The binding site of the anti-HER2 Z_HER2:V2_ affibody molecule contains three arginines, two tyrosines, and one of each: methionine, asparagine, tryptophan, alanine, leucine, asparagine, glutamine, and lysine. A comparison of the lipophilicity using two different scales developed by Kyte and Doolittle (1982) and by Hopp and Woods (1981) suggests that the binding site of Z_IGF1R:4551_ is appreciably more lipophilic than the binding site of Z_HER2:V2_, which may be the reason for the differences in hepatic uptake. This hypothesis is supported by previous findings that elevated hepatic uptake of proteins and peptides is often associated with the presence of “hydrophobic patches” on their surface (Hosseinimehr et al. 2012). Moreover, we have found earlier that the hepatic uptake of anti-HER2 affibody molecules correlated with the hydrophobicity of the binding site (Tolmachev et al. 2012b).

The use of the peptide chelator HEHEHE at the N-terminus led to a decrease in local hydrophobicity and significant suppression of hepatic uptake of [^99m^Tc(CO)_3_]^+^-(HE)_3_-Z_IGF1R:4551_. The use of AEN-sequence at N-terminus of Z _IGF1R:4551_-GGGC is insufficient for this purpose, and the high initial hepatic uptake of ^99m^Tc-Z_IGF1R:4551_-GGGC cannot be compensated by the quick release of radioactivity from liver that results from the non-residualizing properties of the ^99m^Tc-GGGC label. A combination of a hydrophilic tag at the N-terminus and a non-residualizing label at C-terminus might be a possible solution. However, we earlier evaluated a HEHEHE-Z_HER2:342_-GGGC format for labeling of anti-HER2 affibody molecule with ^99m^Tc. It turned out, unfortunately, that ^99m^Tc is also chelated by HEHEHE-tag, and the site specificity of labeling is lost (Lindberg et al. 2012). Other approaches for increasing the hydrophilicity of the affibody molecules are therefore desirable to evaluate.

In conclusion, ^99m^Tc-Z_IGF1R:4551_-GGGC allowed successful visualization of IGF-1R expression in vivo, showing a fast clearance of radioactivity from blood and normal organs and a remarkably low kidney retention. However, its potential in molecular imaging is limited by the high uptake of radioactivity in the liver that might obscure detection of hepatic metastases. The elevated hepatic uptake may be associated with the relatively hydrophobic binding site. This factor should be taken into account in molecular design of affibody molecules and other scaffold proteins.
